# Systemic and specific effects of antihypertensive and lipid-lowering medication on plasma protein biomarkers for cardiovascular diseases

**DOI:** 10.1038/s41598-018-23860-y

**Published:** 2018-04-03

**Authors:** Stefan Enroth, Varun Maturi, Malin Berggrund, Sofia Bosdotter Enroth, Aristidis Moustakas, Åsa Johansson, Ulf Gyllensten

**Affiliations:** 1 Department of Immunology, Genetics, and Pathology, Biomedical Center, Science for Life Laboratory Uppsala University, PO Box 815, SE-75108 Uppsala, Sweden; 2 Department of Medical Biochemistry and Microbiology, Science for Life Laboratory, Uppsala University, PO Box 582, SE-751 23 Uppsala, Sweden; 3 Ludwig Institute for Cancer Research, Science for Life Laboratory, Uppsala University, PO Box 595, SE-751 24 Uppsala, Sweden; 4 Medical Products Agency, PO Box 26, SE-751 03 Uppsala, Sweden

## Abstract

A large fraction of the adult population is on lifelong medication for cardiovascular disorders, but the metabolic consequences are largely unknown. This study determines the effects of common anti-hypertensive and lipid lowering drugs on circulating plasma protein biomarkers. We studied 425 proteins in plasma together with anthropometric and lifestyle variables, and the genetic profile in a cross-sectional cohort. We found 8406 covariate-protein associations, and a two-stage GWAS identified 17253 SNPs to be associated with 109 proteins. By computationally removing variation due to lifestyle and genetic factors, we could determine that medication, per se, affected the abundance levels of 35.7% of the plasma proteins. Medication either affected a single, a few, or a large number of protein, and were found to have a negative or positive influence on known disease pathways and biomarkers. Anti-hypertensive or lipid lowering drugs affected 33.1% of the proteins. Angiotensin-converting enzyme inhibitors showed the strongest lowering effect by decreasing plasma levels of myostatin. Cell-culture experiments showed that angiotensin-converting enzyme inhibitors reducted myostatin RNA levels. Thus, understanding the effects of lifelong medication on the plasma proteome is important both for sharpening the diagnostic precision of protein biomarkers and in disease management.

## Introduction

A large fraction of the human population medicates for chronic diseases such as high blood pressure or high blood lipids. Elevation of blood pressure has been associated with increased cardiovascular morbidity and mortality, including cardiovascular death, myocardial infarction, heart failure and stroke^1^, and is the largest single contributor to worldwide disease burden and mortality^2^ affecting nearly 25% of the adult population of the United States. Clinical management of these diseases involves pharmacotherapy with mono- or combination therapy with Thiazide diuretics, calcium channel blockers, angiotensin-converting enzyme (ACE) inhibitor or angiotensin II receptor blockers, with proven efficacy at reducing blood pressure, but potentially also increasing the risk of cardiovascular events^3–5^. A combination drug therapy generates more synergistic effects that can lower blood pressure, and might result in less severe side effects and improved adherence to a drug regimen. The systemic consequences on human metabolism of long-term drug use for common diseases however remain unknown.

Clinical biomarkers, usually measured in blood plasma, represents an important tool in the diagnosis and follow-up of many common diseases. These biomarkers should ideally only be affected by disease-related factors, but this is rarely the case. For instance, of 145 biomarker candidates for cancer and cardiovascular disease measured in plasma, we previously found that 75% were affected by lifestyle or genetic factors, and that these factors explained between 20–88% of the variation observed in protein abundance between individuals^6,7^. Similarly, non-disease related factors have been shown to affect proteins involved in inflammation and in cerebrospinal fluid^8,9^.

The plasma proteome encompasses proteins originating from a large number of tissues throughout the human body^10^. Mass spectrometry has identified peptides from over 10,288 proteins in plasma^11^, while more strict analyses identified over 3,200 proteins^11^ and up to 1,000 proteins in a single run for one sample^12^. To assess the effect of medication for common diseases, and in particular the effect of antihypertensive and lipid-lowering treatment, on the plasma proteome, we analyzed 425 proteins from 178 KEGG pathways, representing a cross-section of the plasma proteome, in a cross-sectional cohort of over 900 individuals for which detailed data on anthropometrics, lifestyle, use of medication, and genetic variants was known.

## Results

### Analysis of covariates on protein abundance

The proximity extension assay (PEA) was used to study 425 unique proteins in the Northern Swedish Population Health Study (See Methods for details). We first studied the effect of different types of covariates on the plasma levels.

**Figure 1:**
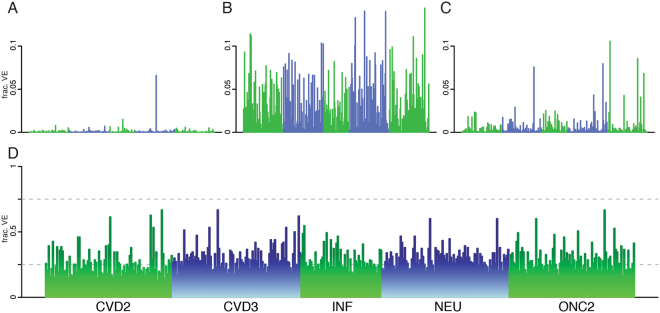
Sources of variation between individuals in abundance of plasma protein variability. Fraction of variance in protein abundance between individuals explained (y-axis) for each protein (x-axis) by use of (**A**) Immunosuppressants (ATC:L04AX), (**B**) Sulfonamides, plain (ATC:C03CA) and, (**C**) Smoking status. Variance explained in the combined model using all available covariates for each of the 425 proteins studied, divided on the five PEA-panels used here; CVD2, CVD3, INF I, NEU and ONC2 (**D**).

Analysis of the 159 anthropometric, lifestyle and clinical covariates showed that 421 proteins had at least one nominally significant association with at least one covariate, and 303 proteins (71.3%) after correction for multiple hypothesis testing (p < 0.05/159/425 = 7.4 × 10^−7^, Table S2). The effect of medication was either very specific (e.g. only one protein was affected, Fig. 1A, Table S3) or very widespread (Fig. 1B). The impact of these covariates on protein levels were of similar effect size, or even larger, than smoking, which is a lifestyle factor that is well-known to influence many biomarkers (Fig. 1C). Since many covariates are dependent, we adjusted for the correlation between covariates using combined modeling of all covariates simultaneously for each protein. The combined models explained between 12.3% and 66.9% of the variance in protein levels between individuals, and this was similar for all the five protein panels used here (Fig. 1D).

### Effect of the genetic variability of plasma protein levels

To identify the genetic effects on plasma protein levels, we performed genome-wide association (GWA) studies, adjusting in the analysis of each protein for any significant covariate (e.g. anthropometric, lifestyle) detected in the combined modeling. The cohort collected in 2006 was used for discovery (n = 663) and the 2009 cohort for replication (n = 240). In the discovery phase, 122 of the 425 proteins showed a significant association with one or many SNPs, and for 109 of these proteins the association remained significant in the replication cohort (Table S4). Analysis of the 109 proteins in the combined data of the 2006 + 2009 cohorts, identified 17253 genome-wide significant associations (Table S5).

**Figure 2:**
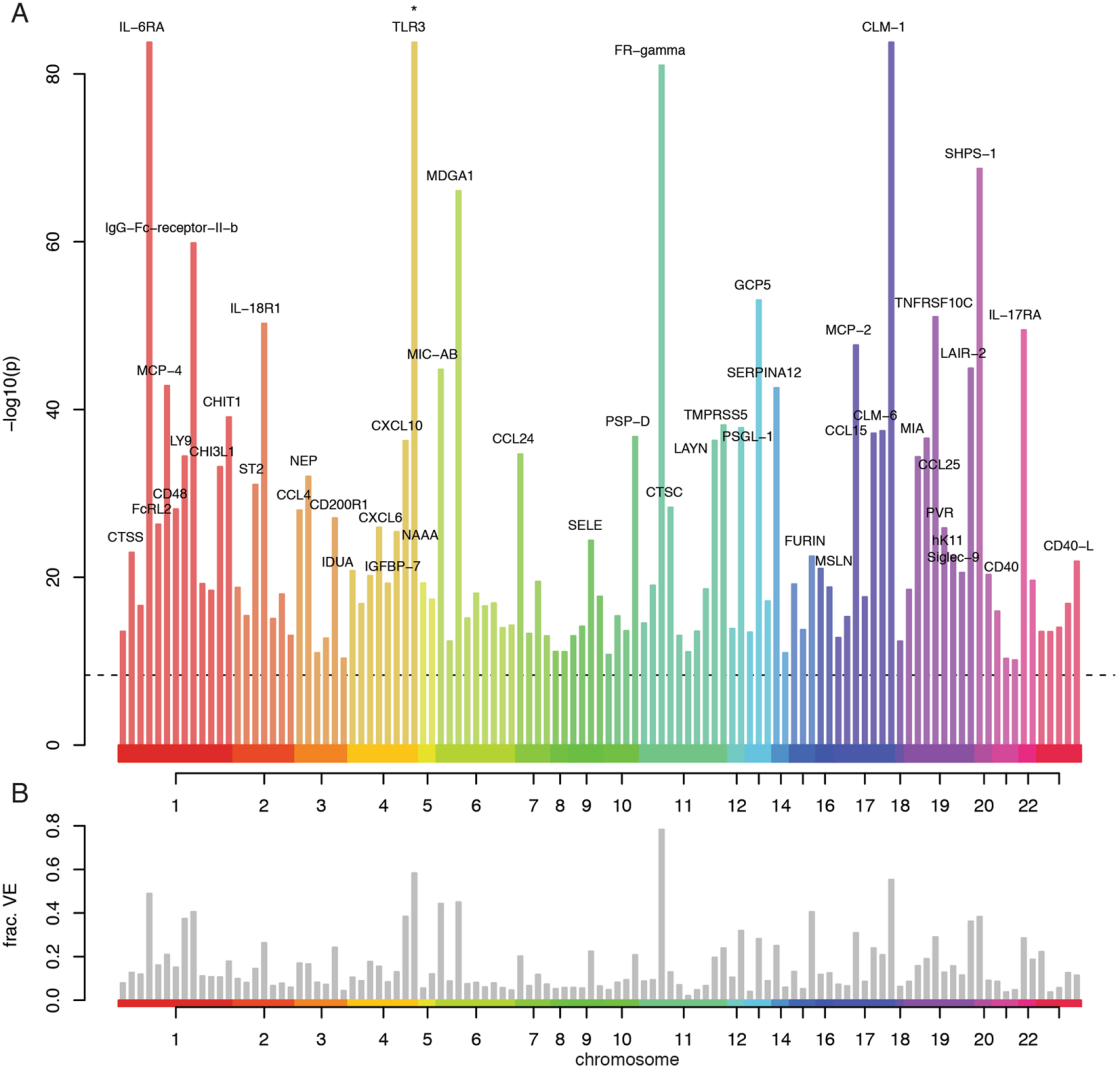
Results of GWAS analysis of the abundance of the 425 plasma proteins. (**A**) P-values for the top markers from the combined analysis for the 109 proteins with replicated hits from the two-stage analysis. Colored bars represent GWAS-hits. Hits with p < 1.0 × 10^−20^ are labeled with protein names. Colors represent chromosomes 1–22 and X. Genome-wide significance (p < 4.79 × 10^−9^) is indicated by a dashed horizontal line. (**B**) Fraction of variance between individuals explained (y-axis) in the raw (unadjusted, untransformed) protein abundance measurements by the top-ranked GWAS hit for each protein.

Genetic associations were found both on the autosomes and the X chromosome (Fig. 2A, Table S5) and 81.7% were located in, or in close proximity (<35 kbp) to, the gene encoding the protein in question. Of the 20 proteins with genetic associations located *in trans*, three were associated with the *ABO*-gene, including a 12-bp deletion (rs8176685) in the first intron that has previously been associated with red blood cell counts^13^. For 101 of the 109 proteins (92.7%) the associated genomic region overlapped with a transcription factor-binding site (ENCODE^14–16^), and for 75 at least one such association was in *cis* with the gene encoding the protein (Table S5). This indicates that for the majority of proteins, the genetic association reflects polymorphisms affecting gene expression. Dosage values for the top-ranking genetic marker was included into models of variance on the abundance levels for each protein. This showed that between 15.4 to 85.2% of the variance between individuals in protein abundance could be explained by the genetic polymorphisms (Fig. 2B).

### Medication effects on the plasma proteome

**Figure 3:**
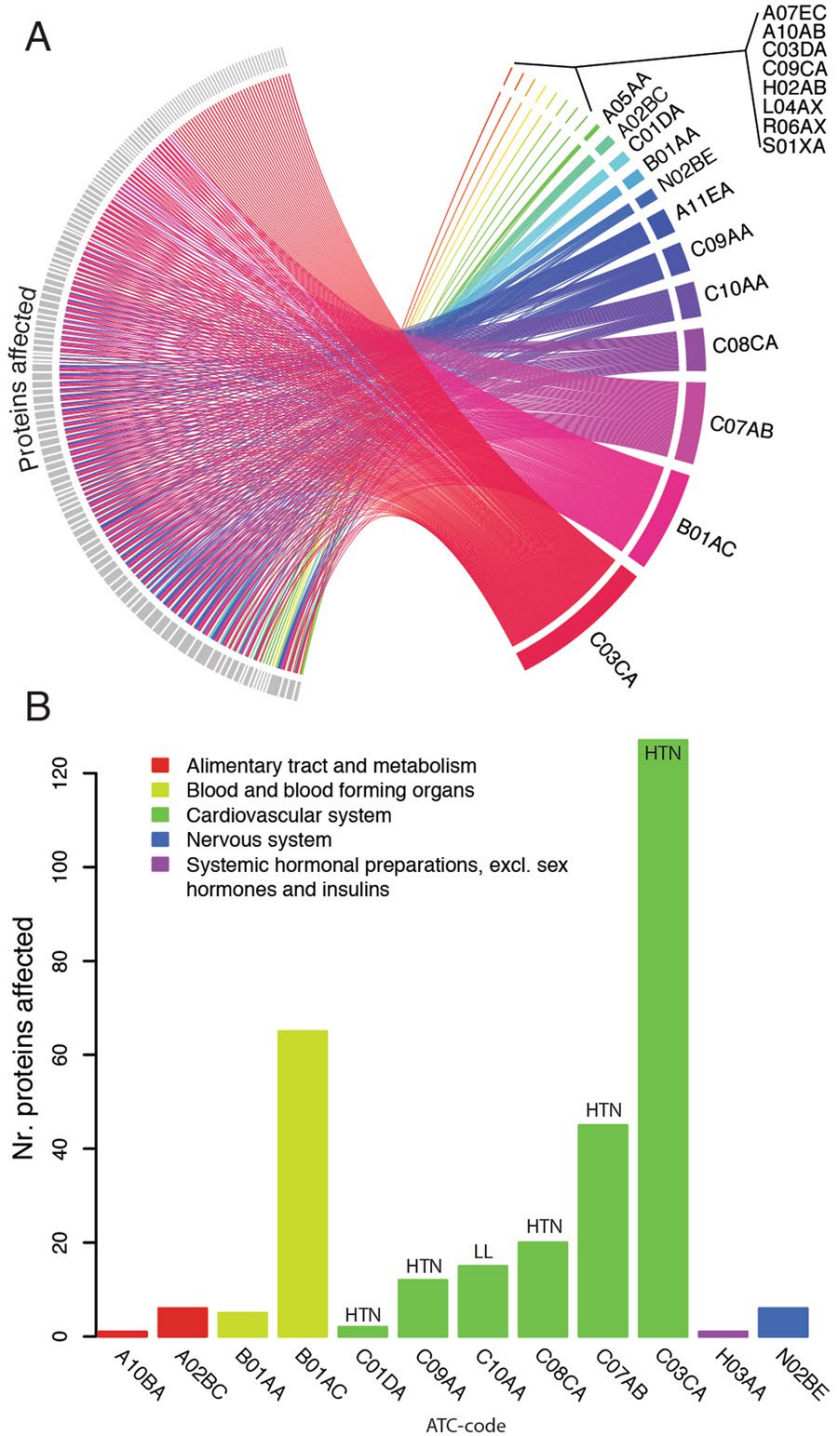
Number of proteins specifically affected by individual drugs. (**A**) Proteins significantly (Wilcoxon-test, Bonferroni adjusted p-value < 0.05) affected by individual drugs without adjustment for additional covariates. Drugs are identified by ATC-codes (Table S3), proteins by grey boxes. Individual associations are given in Table S2. (**B**) Number of proteins (y-axis) affected after computational removal of non-drug related variance. Bars are colored by corresponding first-level ATC-coding. Antihypertensive and lipid-lowering drugs are labeled by ‘HTN’ and ‘LL’ respectively.

Medication as covariate either affected one protein (eight drugs, Table S3) or multiple proteins (12 drugs, 4 to 133 proteins, Fig. 3A, Table S3). Associations with protein levels were found for 20 different drugs, explaining between 2.7 and 14.4% of the variation in abundance levels (Table S2). Plain sulfonamides (ATC:C03CA) showed the highest number of associations and affected 31% (133) of the proteins, followed by platelet aggregation inhibitors (ATC:B01AC), which affected 22% (93 proteins). Since use of medication is not independent of other covariates, we computationally removed variation due to anthropometric, lifestyle and genetic factors to identify the impact of medication. This was performed by using non-users of a medication to model the effects of the covariates, and then adjust the protein abundance in the users as described earlier^7^. For example, use of platelet aggregation inhibitors, excluding heparin, (ATC:B01AC, n = 123), affected 160 proteins before adjustment and 65 after adjustment for other covariates (Fig. 3). C-X-C motif chemokine 10 (CXCL10) had the largest increase in abundance level after adjustment (+0.59 NPX (Normalized Protein eXpression, see Methods for details) after adjustment for weight, smoking status, genetic effects and use of insulin and analogues for injection, fast-acting (ATC:A10AB)). CXCL10 is a pro-inflammatory cytokine that has been reported to increase in mice in response to acetylsalicylic acid which is the active compound in the drugs used in our cohort in the platelet aggregation inhibitors^17^. CXCL10 has also been reported to have a sustained increase at least 16 weeks after experimental induction of myocardial infarction in rats^18^. We then stratified the analysis based on history of myocardial infarction of the individual and use of B01AC, and found that, the levels of CXCL10 were increased in users of B01AC irrespectively of reported history of myocardial infarction (+0.80 NPX, p < 2.9 × 10^−7^ and +0.47 NPX, p < 8.0 × 10^−6^ in the groups with history and no history of myocardial infarction, respectively). This suggests that the drug can result in increased levels of CXCL10, irrespective of previous medical history.

For the 13 types of medication that were used by at least 10 individuals each, we found between 0 and 127 proteins that differed significantly between users and non-users (Fig. 3B, Table S6). There was a correlation (Spearmans’ rho R^2^ = 0.59, p = 3.60 × 10^−3^) between the number of users and the number of associated proteins, and therefore the number of proteins affected by medication is likely to be an underestimate. The most common group of medication in our study cohort was drugs with use within the cardiovascular system, and in that group, the most frequent were lipid-lowering and antihypertensive drugs (Fig. 3B). We therefore performed additional analyses of these two groups.

### Lipid-lowering drugs

Lipid-lowering treatment, including statins, lower circulating LDL-cholesterol levels and protect against major vascular damaging events^19,20^. Statins was the most common lipid-lowering treatment (ATC:C10AA, HMG CoA reductase inhibitors, n = 91) in our cohort. Low- and high-density lipoproteins have previously been measured in NSPHS^21^, and there was no difference in high-density lipoprotein (HDL) cholesterol (two-sided Wilcoxon-ranked based test, p < 0.15) between those on any statin treatment (mean = 1.5 mmol/L) and those not taking lipid-modifying drugs (mean = 1.6 mmol/L). LDL was, however, significantly lower in those medicating (mean = 2.9 mmol/L) compared to controls (mean = 3.6 mmol/L) (two-sided Wilcoxon-ranked based test, p < 2.6 × 10^−9^).

We then built per-protein models using all significant covariates for individuals not using any lipid-modifying drugs (n = 808), and then applied these to individuals on statins (n = 91). Two specific statins (Simvastatin, ATC:C10AA01 and Atorvastatin, ATC:C10AA05) were the two most commonly used statins in our cohort, used by 94.7% (75.3 and 19.4%, respectively, Table S3) of the individuals taking statins. We found no statistical difference (two-sided Wilcoxon-ranked based test, p > 0.05/425 = 1.2 × 10^−4^) between these two groups in protein levels after adjustment for covariates in non-users as described above. The two statin groups were therefore analyzed together, resulting in 104 nominally significant protein associations, and 64 associations after correction for multiple hypothesis testing (False Discovery Rate, Table S7). For 24 of the 64 proteins we found nominally significant correlations (p < 0.05, Spearman’s test, R = −0.18 to 0.51 (Spearman’s Rho)) between LDL-concentration and protein abundance in the controls (Table S7). For these 24 proteins, we cannot separate the effect of medication and lipid levels, and therefore focused on the remaining 40 proteins. For all these 40 proteins, statin-use resulted in higher abundance levels with renin showing the largest increase (+0.40 NPX, p < 3.3 × 10^−7^). Renin is part of the renin-angiotensin system that regulates blood pressure and we therefore analyzed renin in individuals not reporting use of antihypertensive drugs in addition to statins. This included 22 statin users and 674 controls. Similar to using the full set, statin-users had higher abundance levels of renin than non-users (+0.39 NPX, p < 1.7 × 10^−2^), indicating that the effect is related to statin use and not to use of antihypertensive drugs. The second most strongly upregulated protein in statin users was amphiregulin (+0.25 NPX, p < 6.6 × 10^−6^). Amphiregulin levels were adjusted for height only and no correlation was found with either LDL or HDL (R < 1.8 × 10^−2^, p > 0.62).

### Antihypertensive drugs

**Figure 4:**
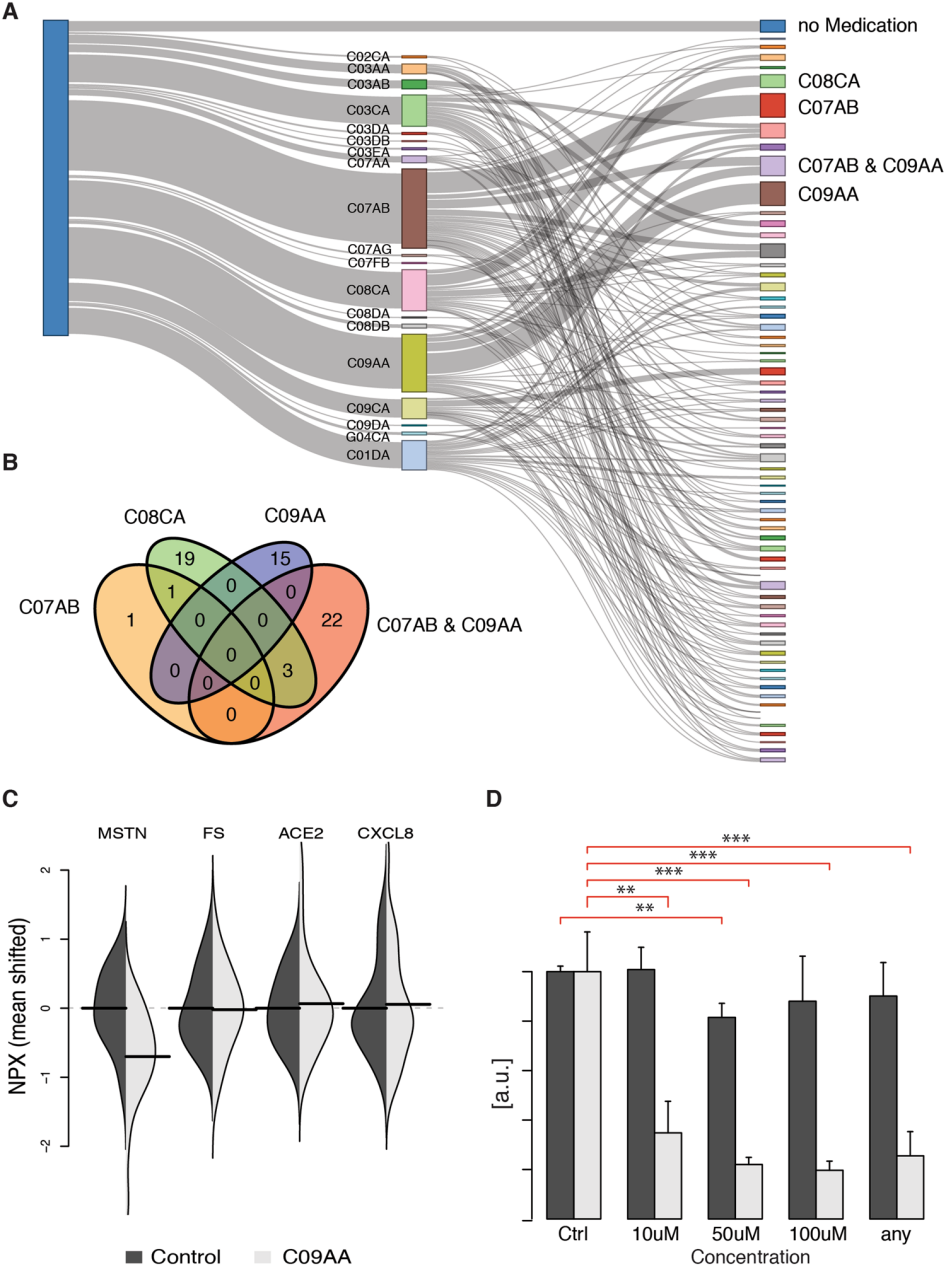
Hypertension drugs and plasma proteins. (**A**) Usage of anti-hypertension drugs. Left-most node contains all individuals (n = 228) identified with hypertension. This group is defined by self-reported diagnosis, self-reported usage of anti-hypertension drugs or with SBP/DBP over 140/90 mmHg. Middle layer shows individual drug-usage and right layer shows combination of drugs used. Groups with more than 10 individuals are labeled in the right-most layer. (**B**) Overlap of significantly different proteins in hypertension individuals on medication compared to individuals without hypertension. For each protein, significantly influencing covariates were detected in individuals not having hypertension and adjusted for in individuals assigned to the hypertension groups. (**C**) Distribution of abundance levels (NPX) for Myostatin (MSTN), Follistatin (FS), Angiotensin-converting enzyme 2 (ACE2) and CXCL8 in medication categories and controls. Non-users are shown in dark grey, users in light grey. Signals have been centered on the mean in controls (non-users). Group mean illustrated by a black horizontal line. (**D**) qPCR-validations on MSTN (light grey) and CXCL8 (dark grey) expression levels in BT-529 upon stimulation with enalapril in concentrations of 10, 50, 100 μM and as a group (‘any’). Red lines indicate significantly (p < 0.05, Student’s t-test, one-sided) different levels in stimulated vs. control. (**Indicate p < 0.01 and ***p < 0.001).

Hypertension is defined as a repeated systolic blood pressure over 140 mmHg and diastolic blood pressure over 90 mmHg. In our cohort 228 individuals were identified with hypertension and they used in total 19 drugs with antihypertensive properties, either as primary or secondary effect (Fig. 4A, middle layer). Medication for hypertension often involve combinations of drugs, and we split the 228 individuals into those using no medication, a single medication, or combinations of two or more drugs (Fig. 4A, right-most layer). Using the 675 individuals not classified with hypertension, we built per-protein models for significant covariates in the control group and applied these models to the hypertension group, including individuals with no medication. We then found 141 nominally significant differences in protein levels between users and non-users of the drugs, out of which 68 (involving 63 proteins) remained significant after correction for multiple hypothesis testing (False Discovery Rate, Table S8). The majority (n = 58) of the proteins associated with hypertension medication represent specific effects of one class, and there was a limited overlap between medication categories (Fig. 4B). The three main types of hypertension drugs, i.e. beta blocking agents (ATC:C07AB), ACE-inhibitors (ATC:C09AA) and dihydropyridine derivatives (ATC:CO8CA), thus result in very different systemic effects on the plasma proteome.

The largest effect of medication increasing the abundance protein of a plasma protein (+1.0 NPX unit) seen was for dihydropyridine derivatives (ATC:CO8CA) on CXCL10. CXCL10 is known to be elevated in patients with untreated hypertension^22^ and is affected by genetic factors^6^. The model we generated in the controls was adjusted for genetic factors only. The largest effect of medication reducing protein abundance (−0.52 NPX) was found for ACE-inhibitors (ATC:C09AA) on myostatin after adjustment for sex and height (Fig. 4C). Proteins of the follistatin-family have been suggested as negative regulators of myostatin^23,24^ but there was no difference in levels of Follistatin (FS) in relation to ACE-inhibitors (Fig. 4C). Similarly, we did not find any difference in levels of Angiotensin-converting enzyme 2 (ACE2), which is the substrate for the ACE-inhibitor enalapril, in users compared to controls (Fig. 4C).

### Enalapril dose-dependently reduces Myostatin RNA-levels

Since the results are based on protein levels in plasma in a cross-sectional cohort, we examined the effect on the RNA-level using cell-culture. The most common substance of ACE-inhibitors in our cohort is enalapril (83.1%, Table S3) followed by cilazapril (10.2%) and ramipril (6.8%), and therefore enalapril was chosen as representative substance. To test the effect of ACE-inhibitors on myostatin levels we studied the effect of enalapril on RNA-levels in the BT549 cell line. The BT549 cell line was chosen because of its native expression of the myostatin encoding gene *MSTN* according to the GOBO-database^25^. BT549 cells were treated with three dosages of enalapril, 10, 50 and 100 μM and *MSTN* expression was quantified by qPCR in technical triplicates in controls (vehicle only) and after 24 h stimuli. A second protein, C-X-C motif chemokine 8 (CXCL8), encoded by the *CXCL8* gene, that did not show any significant change in plasma protein levels in individuals using ACE-inhibitors (p = 0.28, Fig. 4C), was chosen as control. In agreement with the association of medication with the protein level in plasma, *MSTN*-expression was reduced upon administration of enalapril, while expression of *CXCL8* did not change with 10 or 100 μM dosages, but did show a slight reduction at 50 μM (Fig. 4D). The reduction of *MSTN*-expression appears to be dose-dependent with lower levels in higher dosages. In our study cohort, we do not have information on dosages of used medications and we therefore grouped the controls versus any dosage. The *MSTN*-expression was significantly reduced (Student’s t-test, p < 9.0 × 10^−7^), while the reduction in *CXCL8*-expression was non-significant (Student’s t-test, p = 0.12).

## Discussion

The 425 proteins we have studied map into 178 KEGG pathways, representing a cross-section of the plasma proteome^11^. Our conclusions are therefore likely to reflect general effects on the plasma proteome variability. Consistent with previous studies, a majority of the proteins are affected by anthropometric, lifestyle or genetic factors, and in combination these explained up to 85.6% of the observed intra-individual variance. The NSPHS-cohort comprises just over one thousand individuals and a recent study^26^ based on over three thousand individuals found genetic associations for 58 out of 83 (67.5%) plasma proteins studied. Thus, the number of associations found here is likely to be an underestimate. Relative to anthropometric, lifestyle and genetic factors, the impact of common medication is largely unknown. Our analysis has revealed important findings.

First, long-term medication can have very specific effects on a single protein or very wide-spread effects on the plasma proteome, indicative of systemic effects with unknown consequences. One of the proteins showing the largest difference in abundance between users and non-users of lipid-lowering statins was amphiregulin. Amphiregulin is an epidermal growth factor family member is involved in the proliferation, migration and apoptosis of cells^27^. Chronically elevated levels of amphiregulin have been reported in inflammatory diseases and cancer^27^, and antibody based depletion of amphiregulin has been shown to inhibit ovarian tumor growth^28^. Thus, the increased circulating levels of amphiregulin following statin treatment are of concern.

Comparison between individuals that are on or off medication for hypertension shows that, in general, different medications affect specific sets of proteins. Many of the proteins affected by these drugs have not previously been linked to hypertension, indicating that many downstream effects of medication on the proteome have yet to be discovered. One example is the strong effect of ACE-inhibitors on lowering of myostatin levels. Myostatin is a negative regulator of skeletal muscle growth and inhibition of myostatin leads to increased muscle mass in mice^29^. Use of ACE-inhibitors in elderly humans has been shown to slow decline in muscle strength and improve walking speeds^30,31^ and improvements of muscle power^32^ has been observed in antibody-based inhibition of myostatin in treatment of elderly weak fallers. Thus, both observational and molecular studies are suggestive of a connection between use of ACE-inhibitors and improvements in muscle traits in human, and our results indicate that this could be mediated by a down-regulation of myostatin expression.

Second, the size effect of some medications on proteome variability is of the same magnitude of previously recognized covariates, such as age or smoking status. To specifically isolate the effects of medication, we adjusted for other significant covariates among non-users, which reduced the number of differences between users and non-users by 63%, from a total of 853 to 312. Even after adjusting for other covariates, the effect of medication is substantial, and explains up to 14.4% of proteome variability. This implies that in epidemiological studies and in utilizing plasma protein biomarkers, the effect of common medication has to be acknowledged and included in the analysis. Fortunately, given that information on medication is collected, the effect on protein levels can be taken into account, determining individual normal thresholds for plasma protein-based clinical indicators. This will result in a better precision of the protein biomarkers as clinical indicators and more efficient identification of novel biomarkers^7^.

The present study has some limitations. The NSPHS is a cross-sectional cohort, and the samples were collected at a single time-point. A longitudinal study including measurements before and after introduction of the drug under study, would have enabled monitoring of the effect of the drug on disease development and protein levels. However, we were able to show that the effect discovered here by ACE-inhibitors on circulating myostatin levels can be replicated under controlled conditions on RNA-level. The proteins investigated here is not the complete set of proteins present in plasma, nor a random selection, but instead determined by the available multiplex PEA panels. The selection bias limits the possibility to perform downstream functional analysis of proteins that changes in users of a specific drug, such as pathway enrichment or functional classification, using e.g. Gene Ontology-analysis. Although a large number of measurements of phenotypes have been performed in NSPHS, we lack several phenotypes that would have allowed us to investigate relations between protein biomarkers and underlying functional factors contributing to development of disease, such as risk factors for metabolic syndrome^33^, components of the adrenergic system^34^ and components involved in the regulation of the renin-angiotensin-aldosterone system^35^ in relation to blood pressure, pre-hypertension or hypertension. The foremost strength of our study is the large number of phenotypes characterized in the NSPHS cohort, including the data on the genetic constitution. These allow us to adjust for non-disease related variance in the comparisons between user and non-users of specific drugs.

In conclusion, we have shown that common medication induces a large number of effects on plasma proteins and biomarkers, most of which have not been previously recognized or predicted. Some of these consequences on the plasma proteome represent have positive or negative secondary effects that may interfere with the purpose of the medication. The results underscore the need to identify of the network of medication-caused proteome effects using patient cohorts subjected to specific medications, in order to fully understand the metabolic consequences of common chronic medications.

## Methods

### Samples

The Northern Sweden Population Health Study (NSPHS) was initiated in 2006 as a health survey in the county of Norrbotten, Sweden, to study the medical consequences of lifestyle and genetic factors. The first phase (2006) included 719 individuals (KA06 cohort) and a second phase (2009) another 350 individuals (KA09 cohort). For each participant, serum and plasma were collected and stored at −70 °C on site. DNA has been extracted for genetic analyses and detailed descriptions of this study have been previously published^36–38^. A questionnaire was used to collect data on medication and lifestyle. Blood groups according to the ABO-system were assigned based on four SNPs as previously described^6^. Around 15% of the participants adhered to a traditional lifestyle (TLS) involving reindeer herding and crafts. Differences in diet between this group and those with a lifestyle typical of industrialized regions have been shown to increase levels of circulating blood lipids^39^ which motivates to include the TLS adherence as a covariate. Sample-round was also included as a covariate since storage-time affects the plasma protein concentrations^40^. Here, 903 samples (KA06/KA09, n = 663/240) were included.

### Coding of medication

Use of medication was investigated using a questionnaire and annotated using the Anatomical Therapeutic Chemical (ATC) classification system. Use of medication was coded as yes/no, since no dosage information was available. The most commonly used were: B01AC (platelet aggregation inhibitors excl. heparin, n = 106), C10AA (HMG CoA reductase inhibitors, n = 91), C07AB (beta blocking agents, selective, n = 81), C09AA (ACE inhibitors, plain, n = 59), C08CA (dihydropyridine derivatives, n = 42), H03AA (thyroid hormones, n = 38), C03CA (sulfonamides, plain, n = 32), C01DA (organic nitrates, n = 30), N02BE (anilides, n = 29), A02BC (proton pump inhibitors, n = 28), R03AK (Inhaled adrenergics in combination with corticosteroids or other drugs, excl. anticholinergics, n = 23), R03BA (Inhaled glucocorticoids, n = 22), A10BA (biguanides, n = 21) and C09CA (angiotensin II antagonists, plain, n = 21). A full account of the ATC classifications used here including distributions of individual drugs are listed in Table S3.

### Ethical considerations

The NSPHS study was approved by the Regional Ethics Committee in Uppsala in compliance with the Declaration of Helsinki^41^ (Regionala Etikprövningsnämnden, Uppsala, Dnr. 2005:325 with approval of extended project period on 2016-03-19). All participants gave their written informed consent to the study including the examination of environmental and genetic causes of disease. In cases where the participant was not of age, a legal guardian signed additionally.

### Plasma protein profiles

We used the Proximity Extension Assay (PEA)^42^ to measure protein abundance. This is an affinity-based assay and for each protein, a pair of oligonucleotide-labeled antibody probes bind to the targeted protein and if the two probes are in close proximity, a PCR target sequence is formed by a proximity-dependent DNA polymerization event and the resulting sequence is subsequently detected and quantified using real-time PCR. The resulting abundance levels are given in NPX (Normalized Protein eXpression). Each proximity extension assay has a lower detection limit calculated based on negative controls that are included in each run and measurements below this limit were removed from further analysis. Assay characteristics including detection limits, assay performance and validations are available from the manufacturer (www.olink.com).

Here, the abundance levels of 441 proteins in plasma were analyzed by the Proximity Extension Assay (PEA)^42^ using five Olink Proseek Multiplex panels (CVD II, CVD III, INF I, ONC 2 and NEU I, www.olink.com) and quantified by real-time PCR using the Fluidigm BioMark™ HD real-time PCR platform^42^. PEA gives abundance levels in NPX (Normalized Protein eXpression). Three proteins were read out in duplicates on multiple panels (interleukin 6 (IL-6), interleukin 12 (IL-12) and stem cell factor (SCF)) and these were highly correlated between runs (p < 7 × 10^−209^, Spearman’s rho R^2^ > 0.67). The assays have been selected based on evidence of a relation to disease and not all individuals in a cross-sectional cohort may have abundance levels above LOD (limit of detection). We removed 16 proteins that had less than 20% of measurements above LOD and the final analysis was based on 425 unique proteins.

### Genetic data

The NSPHS cohort have previously been genotyped^43^ on the Illumina Infinium HapMap300v2 BeadChip (KA06) or Illumina Human OmniExpress BeadChip (KA09). Both sub-cohorts have also been genotyped^6^ on the Illumina Human Exome Beadchip. Due to differences in genotyping platform, the two cohorts were here imputed separately using IMPUTE2(v2.3.2) with a pre-phasing approach^44^. The input data were phased chromosome-wise using SHAPEIT version 2.5(r790). The reference panel used was the autosomal 1000 Genomes Phase3 integrated haplotypes (released in October 2014) and the X-chromosome Genomes Phase3 integrated haplotypes (released in August 2015) accessed from the IMPUTE Web resource^45^. IMPUTE2 was run with default parameters. Data were imputed in chunks of around 5 M base pairs ensuring ≥200 genotyped SNPs in each chunk. The resulting data were filtered on marker level by requiring IMPUTE’s ‘info’ score > 0.3 in both cohorts before merging with GTOOL(v0.7.5)^46^ requiring a dosage threshold above 0.9 in at least 95% of the individuals. The merged data were filtered using QCTOOL (v1.4)^47^ requiring a Bonferroni corrected Hardy-Weinberg cut-off of 0.05 and a minor-allele frequency corresponding to at least one chromosome in the whole material. The final data included 10,442,416 SNPs and INDELs on chromosomes 1–22, M and X.

### Availability of data and material

The detailed proteomic, genetic, anthropometric and lifestyle individual raw data used in this study together with the origin of the cohort would compromise individual privacy if the data was made publically available. The datasets generated and analyzed during the current study are therefore not publicly available but can be made available from the corresponding author on reasonable request.

### Cell culture and qPCR validations

Human breast cancer BT-549 cells were cultured in RPMI-1640 with 10% fetal bovine serum and penicillin-streptomycin, and were treated with water (vehicle) or enalapril (Sigma Aldrich, #CDS020548) for 24 h at the indicated concentrations. Total RNA was extracted using TRIzol (Invitrogen, #15596026). Complementary DNA was prepared using the iScript cDNA synthesis kit (Bio-Rad Laboratories AB, Solna, Sweden). Real-time PCR was done using the KAPA kit (SIGMA ALDRICH #KK4608) using the following primers: human *HPRT1* forward 5′-CCCTGGCGTCGTGATTAGT-3′ reverse 5′-CACCCTTTCCAAATCCTCAGC-3′, human *MSTN* forward 5′-TGGCTCAAACAACCTGAATCCA-3′ reverse 5′-GGATCTTTTTGGTGTGTCTGTTACCTT-3′, human *CXCL8* forward 5′-CTCTTGGCAGCCTTCCTGAT-3′ reverse 5′-TCCACTCTCAATCACTCTCAGTTCT-3′.

### Statistical analysis and Figures

All calculations were carried out in R^48^ (version 3.2.3) or perl using custom scripts. PEA-signals were first normalized by plate number and sampling round using the MDimNormn-package^49^. Significance levels for influencing variables were calculated separately for each protein by fitting a generalized linear model using the’glm’ function including each covariate separately and simultaneously. The significance of each independent covariate’s contribution to the total variance was taken directly from the model and for the combined model it was estimated using an ANOVA-approach as implemented in the ‘anova’ function on the resulting generalized linear model. Covariates were considered significant for a specific protein if their Bonferroni-adjusted *p*-values were below 0.05. Fraction of variance explained was calculated from the models using the Cox & Snell-method as implemented in the modEvA-package^50^. For the GWAS, each PEA-signal was adjusted for significant covariates from the combined models and rank-transformed to normality by using the ‘rntransform’ function from the R-package GenABEL(v1.8.0)^51^. The NSPHS includes related individuals and special care has to be taken to avoid relational biases. Therefore, all genetic association calculations were carried out as described earlier^6^ using the GenABEL or ProbABEL (‘palinear’, version 0.4.5) software suites^51^, which have been developed to enable analyses of related individuals. The KA06 cohort was used as discovery cohort and KA09 as replication cohort. Strict Bonferroni-adjusted *p*-values (*p*-value < 4.79 × 10^−9^, 0.05/10,442,416) were used to report significance in the discovery cohort and the replication cohort (*p*-value < 0.05/number of significant SNPs in the discovery cohort). Annotations of GWAS-hits in relation to genes were done using ANNOVAR^52^. Overlaps with the ENCODE transcription factor binding sites^14–16^ were analysed with BEDTools^53^ (v2.25.0). Correlations were calculated using Spearman’s rho. Beanplots were drawn using the ‘beanplot’ package^54^, the circular charts using the ‘circlize’-package^55^ and the Sankey-diagram using the ‘rCharts’-package (https://github.com/ramnathv/rCharts). All other figures were produced using custom in-house R-scripts.

## Electronic supplementary material

Supplementary Tables 1-8

## References

[1] Rosendorff C (2015). Treatment of Hypertension in Patients With Coronary Artery Disease: A Scientific Statement from the American Heart Association, American College of Cardiology, and American Society of Hypertension. Journal of the American College of Cardiology.

[2] Poulter NR, Prabhakaran D, Caulfield M (2015). Hypertension. Lancet.

[3] Dunder K, Lind L, Zethelius B, Berglund L, Lithell H (2003). Increase in blood glucose concentration during antihypertensive treatment as a predictor of myocardial infarction: population based cohort study. BMJ.

[4] Psaty BM (1995). The risk of myocardial infarction associated with antihypertensive drug therapies. JAMA: the journal of the American Medical Association.

[5] Brouwers FM (2014). Beta-blockers are associated with increased risk of first cardiovascular events in non-diabetic hypertensive elderly patients. Pharmacoepidemiol Drug Saf.

[6] Enroth S, Johansson A, Enroth SB, Gyllensten U (2014). Strong effects of genetic and lifestyle factors on biomarker variation and use of personalized cutoffs. Nat Commun.

[7] Enroth S, Bosdotter Enroth S, Johansson A, Gyllensten U (2015). Effect of genetic and environmental factors on protein biomarkers for common non-communicable disease and use of personally normalized plasma protein profiles (PNPPP). Biomarkers.

[8] Larsson A (2015). The body mass index (BMI) is significantly correlated with levels of cytokines and chemokines in cerebrospinal fluid. Cytokine.

[9] Larsson A (2015). The effects of age and gender on plasma levels of 63 cytokines. J Immunol Methods.

[10] Zhang H (2007). Mass spectrometric detection of tissue proteins in plasma. Mol Cell Proteomics.

[11] Nanjappa V (2014). Plasma Proteome Database as a resource for proteomics research: 2014 update. Nucleic Acids Res.

[12] Geyer PE (2016). Plasma Proteome Profiling to Assess Human Health and Disease. Cell Syst.

[13] McLachlan S (2016). Replication and Characterization of Association between ABO SNPs and Red Blood Cell Traits by Meta-Analysis in Europeans. PLoS One.

[14] Wang J (2013). Factorbook.org: a Wiki-based database for transcription factor-binding data generated by the ENCODE consortium. Nucleic Acids Res.

[15] Wang J (2012). Sequence features and chromatin structure around the genomic regions bound by 119 human transcription factors. Genome Res.

[16] Gerstein MB (2012). Architecture of the human regulatory network derived from ENCODE data. Nature.

[17] Fujita M (2011). COX-2 blockade suppresses gliomagenesis by inhibiting myeloid-derived suppressor cells. Cancer Res.

[18] Altara R (2016). CXCL10 Is a Circulating Inflammatory Marker in Patients with Advanced Heart Failure: a Pilot Study. J Cardiovasc Transl Res.

[19] Cholesterol Treatment Trialists C (2010). Efficacy and safety of more intensive lowering of LDL cholesterol: a meta-analysis of data from 170,000 participants in 26 randomised trials. Lancet.

[20] Herrington W, Lacey B, Sherliker P, Armitage J, Lewington S (2016). Epidemiology of Atherosclerosis and the Potential to Reduce the Global Burden of Atherothrombotic Disease. Circ Res.

[21] Aulchenko YS (2009). Loci influencing lipid levels and coronary heart disease risk in 16 European population cohorts. Nature genetics.

[22] Antonelli A (2012). High serum levels of CXC (CXCL10) and CC (CCL2) chemokines in untreated essential hypertension. International journal of immunopathology and pharmacology.

[23] Hill JJ (2002). The myostatin propeptide and the follistatin-related gene are inhibitory binding proteins of myostatin in normal serum. J Biol Chem.

[24] Amthor H (2004). Follistatin complexes Myostatin and antagonises Myostatin-mediated inhibition of myogenesis. Dev Biol.

[25] Ringner M, Fredlund E, Hakkinen J, Borg A, Staaf J (2011). GOBO: gene expression-based outcome for breast cancer online. PLoS One.

[26] Folkersen L (2017). Mapping of 79 loci for 83 plasma protein biomarkers in cardiovascular disease. PLoS Genet.

[27] Berasain C, Avila MA (2014). Amphiregulin. Semin Cell Dev Biol.

[28] Carvalho S (2016). An antibody to amphiregulin, an abundant growth factor in patients’ fluids, inhibits ovarian tumors. Oncogene.

[29] Lee SJ, McPherron AC (2001). Regulation of myostatin activity and muscle growth. Proc Natl Acad Sci USA.

[30] Campins L (2017). Oral Drugs Related with Muscle Wasting and Sarcopenia. A Review. Pharmacology.

[31] Onder G (2002). Relation between use of angiotensin-converting enzyme inhibitors and muscle strength and physical function in older women: an observational study. Lancet.

[32] Becker C (2015). Myostatin antibody (LY2495655) in older weak fallers: a proof-of-concept, randomised, phase 2 trial. Lancet Diabetes Endocrinol.

[33] Tune JD, Goodwill AG, Sassoon DJ, Mather KJ (2017). Cardiovascular consequences of metabolic syndrome. Transl Res.

[34] Ciccarelli M, Santulli G, Pascale V, Trimarco B, Iaccarino G (2013). Adrenergic receptors and metabolism: role in development of cardiovascular disease. Front Physiol.

[35] Kachur S, Morera R, De Schutter A, Lavie CJ (2018). Cardiovascular Risk in Patients with Prehypertension and the Metabolic Syndrome. Curr Hypertens Rep.

[36] Johansson A (2009). Common variants in the JAZF1 gene associated with height identified by linkage and genome-wide association analysis. Hum Mol Genet.

[37] Igl W, Johansson A, Gyllensten U (2010). The Northern Swedish Population Health Study (NSPHS)–a paradigmatic study in a rural population combining community health and basic research. Rural and remote health.

[38] Enroth, S., Dahlbom, I., Hansson, T., Johansson, A. & Gyllensten, U. Prevalence and sensitization of atopic allergy and coeliac disease in the Northern Sweden Population Health Study. *International journal of circumpolar health* **72**, 10.3402/ijch.v72i0.21403 (2013). 10.3402/ijch.v72i0.21403 PMC3754550 23986895

[39] Igl, W. *et al*. Animal source food intake and association with blood cholesterol, glycerophospholipids and sphingolipids in a northern Swedish population. *International journal of circumpolar health* **72**, 10.3402/ijch.v72i0.21162 (2013). 10.3402/ijch.v72i0.21162 PMC3753141 23984293

[40] Enroth, S., Hallmans, G., Grankvist, K. & Gyllensten, U. Effects of Long-Term Storage Time and Original Sampling Month on Biobank Plasma Protein Concentrations. *EBioMedicine*, **12**, 309-314 10.1016/j.ebiom.2016.08.038. 10.1016/j.ebiom.2016.08.038 PMC5078583 27596149

[41] World Medical Association Declaration of Helsinki: ethical principles for medical research involving human subjects. *JAMA: the journal of the American Medical Association* **284**, 3043–3045 (2000). 11122593

[42] Assarsson E (2014). Homogenous 96-Plex PEA Immunoassay Exhibiting High Sensitivity, Specificity, and Excellent Scalability. PLoS ONE.

[43] Johansson A (2013). Identification of genetic variants influencing the human plasma proteome. Proc Natl Acad Sci USA.

[44] Howie B, Fuchsberger C, Stephens M, Marchini J, Abecasis GR (2012). Fast and accurate genotype imputation in genome-wide association studies through pre-phasing. Nature genetics.

[45] IMPUTE2 reference panels. https://mathgen.stats.ox.ac.uk/impute/1000GP_Phase3.html, http://mathgen.stats.ox.ac.uk/impute/impute_v2.html-reference (2015).

[46] Freeman, C. & Marchini, J. *GTOOL*, http://www.well.ox.ac.uk/~cfreeman/software/gwas/gtool.html (2015).

[47] Band, G. & Marchini, J. *QCTOOL*, http://www.well.ox.ac.uk/~gav/qctool/, http://www.well.ox.ac.uk/~gav/qctool/ (2015).

[48] R Develpment Core Team. *R: A language and environment for statistical computing*., (R Foundation for Statistical Computing, 2015).

[49] Hong MG, Lee W, Nilsson P, Pawitan Y, Schwenk JM (2016). Multidimensional Normalization to Minimize Plate Effects of Suspension Bead Array Data. J Proteome Res.

[50] Model Evaluation and Analysis. v. R-package version 1.2.8 build r99 (https://r-forge.r-project.org/projects/modeva/, 2016).

[51] Aulchenko YS, Ripke S, Isaacs A, Van Duijn CM (2007). GenABEL: an R library for genome-wide association analysis. Bioinformatics.

[52] Wang K, Li M, Hakonarson H (2010). ANNOVAR: functional annotation of genetic variants from high-throughput sequencing data. Nucleic Acids Res.

[53] Quinlan AR, Hall IM (2010). BEDTools: a flexible suite of utilities for comparing genomic features. Bioinformatics.

[54] Kampstra P (2008). Beanplot: A Boxplot Alternative for Visual Comparison of Distributions. Journal of Statistical Software.

[55] Gu Z, Gu L, Eils R, Schlesner M, Brors B (2014). circlize Implements and enhances circular visualization in R. Bioinformatics.

